# ^1^H-NMR Analysis of Wine Metabolites: Method Development and Validation

**DOI:** 10.3390/molecules31010065

**Published:** 2025-12-24

**Authors:** Guillaume Leleu, Rémi Butelle, Daniel Jacob, Lou-Ann Kurkiewicz, Jean-Claude Boulet, Catherine Deborde, Matthieu Dubernet, Laetitia Gaillard, Antoine Galvan, Karen Gaudin, Alexandra Gossé, Markus Herderich, Annick Moing, Sophie Rosset, Flynn Watson, Gregory Da Costa, Tristan Richard

**Affiliations:** 1 Univ. Bordeaux, Bordeaux INP, INRAE, OENO, UMR 1366, ISVV, F-33140 Villenave d’Ornon, France; guillaume.leleu@u-bordeaux.fr (G.L.); remi.butelle@u-bordeaux.fr (R.B.); lou-ann.kurkiewicz@u-bordeaux.fr (L.-A.K.); karen.gaudin@u-bordeaux.fr (K.G.); gregory.da-costa@u-bordeaux.fr (G.D.C.); 2 INRAE, Univ. Bordeaux, Biologie du Fruit et Pathologie, UMR 1332, Centre INRAE de Nouvelle Aquitaine Bordeaux, F-33140 Villenave d’Ornon, France; daniel.jacob@inrae.fr (D.J.); catherine.deborde@inrae.fr (C.D.); annick.moing@inrae.fr (A.M.); 3 INRAE, CALIS/PROBE Research Infrastructures, BIBS Facility, F-44300 Nantes, France; 4 INRAE, Institut Agro Montpellier, Univ. Montpellier, SPO, UMR 1083, F-34060 Montpellier, France; jean-claude.boulet@inrae.fr; 5 Laboratoire Dubernet, 35 Rue de Combe du Meunier, 11100 Montredon-des-Corbières, France; matthieu.dubernet@dubernet.com; 6 Service Commun des Laboratoires, 3 Avenue du Dr. Albert Schweitzer, F-33600 Pessac, France; laetitia.gaillard@scl.finances.gouv.fr (L.G.); antoine.galvan@scl.finances.gouv.fr (A.G.); sophie.rosset@scl.finances.gouv.fr (S.R.); 7 Les Grands Chais de France, 1 Rue de la Division Leclerc, F-67290 Petersbach, France; alexandra.gosse@lgcf.fr; 8 The University of Adelaide, Waite Research Institute, Urrbrae, SA 5064, Australia; markus.herderich@awri.com.au; 9 The Australian Wine Research Institute, P.O. Box 46, Glenside, SA 5065, Australia; flynn.watson@awri.com.au; 10 Metabolomics Australia, P.O. Box 46, Glenside, SA 5065, Australia

**Keywords:** wine, metabolomics, ^1^H-NMR, quantification, method validation

## Abstract

Wine, as a high-value product, is vulnerable to counterfeiting. To tackle increasingly sophisticated fraud, innovative analytical approaches are required. However, they must undergo rigorous validation. Proton nuclear magnetic resonance spectroscopy (^1^H-NMR) is intrinsically quantitative, reproducible, and fast, making it a promising tool for official control. This study presents the development and validation of a standardised and fully automated workflow for the quantification of 20 oenologically relevant compounds, including organic acids, sugars, alcohols, esters, phenolics, and an alkaloid. The method combines optimised sample preparation, external quantification standards, spectrometer calibration, and a dedicated R package (RnmrQuant1D) for fully automated spectral processing, enabling high-throughput and operator-independent analysis. Validation was performed under intermediate precision according to OIV metrological standards, evaluating accuracy, precision, robustness, limits of quantification, and measurement uncertainty. The results demonstrated excellent linearity, trueness, and reproducibility, matching the targeted analytical performance. Measurement uncertainties were estimated both by conventional linear modelling and by a dynamic approach better suited to detection limits. The workflow is easy to implement, requires minimal sample consumption, and substantially reduces operator bias. Beyond validating a robust method, this study provides a framework for harmonised, transferable ^1^H-NMR workflows that could support large-scale databases, integration with chemometric models, and ultimately, ^1^H-NMR’s recognition as a relevant method for wine authentication and quality control. This work fills a crucial gap in wine analysis by uniting practical application and rigorous methods, enabling broader adoption in control laboratories worldwide.

## 1. Introduction

Wine occupies a central place in the global agri-food sector, with a global consumption of 214 million hectolitres, including 100 million hectolitres—valued at around €36 billion—traded in 2024 between international markets [1]. Wine ranks third among alcoholic beverages worldwide, accounting for 12% of global consumption after spirits (45%) and beer (35%) [2]. In addition to their substantial economic contributions, grape and wine production also have significant socio-cultural impact, with several wine-growing landscapes listed as UNESCO World Heritage Sites. Given its economic importance, wine is particularly vulnerable to fraud, such as adulteration with the use of non-permitted additives or counterfeiting with cheaper products or relabelling of inferior products to imitate more prestigious brands. Overall, wine fraud is estimated to result in an average direct loss in sales of €500 million per year in Europe alone [3]. To address these increasingly sophisticated and difficult-to-detect frauds, the use of advanced analytical methods is essential.

Various analytical techniques for quality control and the detection of potential fraud are currently used by competent laboratories and recognised by the International Organisation of Vine and Wine (OIV) for wine analysis in the Compendium of International Methods of Analysis [4]. These include chromatographic, spectroscopic, and isotopic approaches, some of which, such as isotope ratio mass spectrometry and site-specific natural isotope fractionation (SNIF) NMR, have already been incorporated into collective European initiatives such as the EU Wine Data Bank for the fight against fraud [5,6]. In recent years, proton nuclear magnetic resonance spectroscopy (^1^H-NMR) has emerged as a particularly relevant approach for wine analysis [7,8]. Its intrinsic quantitative nature, linearity, reproducibility, and high throughput due to minimal sample preparation requirements and short measurement times, combined with its ability to simultaneously quantify several different compounds from small sample volumes, offer clear advantages for addressing the complexity of wine fraud [5,9,10].

At the experimental scale, ^1^H-NMR analysis of wine metabolites has already yielded promising results, though its integration into official standards for wine control remains limited to date. The work of Godelmann et al., who validated the quantification of six compounds in wines using ^1^H-NMR, namely glucose, malic acid, acetic acid, fumaric acid, shikimic acid, and sorbic acid, led to the initial recognition of this analytical tool by the OIV as a provisional method for analysis of wine (Type IV method OIV-MA-AS316-01) [11,12]. More recently, studies focusing on quantifying a single compound have added to published work with promising leads, such as ethanol quantification [13]. In addition, proline quantification has also been developed up to the validation stage [14]. However, rigorous validation of a standardised and transferable analytical method covering an expanded range of compounds remains necessary.

This study builds upon previous work and aims to bring about substantial improvements in the analysis of wines using ^1^H-NMR. The approach described in this publication quantifies 20 ubiquitous wine compounds using a standardised, open, and transferable method. The objective is to enhance NMR’s recognition by the OIV for wine analysis and thus facilitate its integration into official methods for wine control. The selected compounds were chosen based on various criteria, including their relevance to oenology and their ability to be analysed using a fully automated process. Additional compounds may be added later, particularly using information extracted from J-resolved sequences. The proposed workflow covers the entire analytical process: from preparation of samples and quantification standards, calibration of the measuring instrument, and acquisition of spectra to fully automated data processing for the quantification of the selected compounds belonging to different chemical classes. By minimising the potential for operator-related error and reducing the need for advanced NMR expertise, the method is designed to ensure broad acceptance and better reproducibility across laboratories.

After each step of the proposed workflow is discussed [15], the results of the evaluation of the method’s validity parameters under intermediate precision conditions are presented. In accordance with the OIV standard OIV-OENO 418-2013 [16] on the validation of analytical methods, trueness, precision, accuracy, and robustness were assessed against pre-set metrological objectives [17]. In addition, measurement uncertainty and quantification limits are determined using a traditional approach and through dynamic uncertainty modelling to provide a comprehensive assessment of the method’s performance [16,18].

## 2. Results and Discussions

### 2.1. From Sample Preparation to Data Extraction

#### 2.1.1. Sample Preparation Steps and ^1^H-NMR Acquisitions

Numerous methods for the preparation of wine have been proposed in the literature [9]. In our method, an initial centrifugation step reduces the impact of solid particles present in the wine, which could affect the ^1^H-NMR acquisition of the sample [9]. The temperature for centrifugation is set at 15 °C to avoid heating of the sample. The pH of the samples was set at 3.10 using a buffer solution, followed by automated pH adjustment. This adjustment aims to minimise peak shift in the resultant ^1^H-NMR spectra [11,19,20]. Wines span a wide range of pH values due to high levels of diverse organic acids and are thus resistant to buffering effects [21]. Different buffer recipes have been tested to achieve a pH as close to 3.10 as possible after addition. However, in all cases, pH adjustment is required. Furthermore, the buffer solution is prepared directly in the deuterated solvent, which reduces dilution effects during sample preparation, maximising the sensitivity of wine components. Deuterated solvent is required in the solution to allow ‘locking’ of the sample.

Wine is mainly composed of water and ethanol; thus, suppression of these signals is essential to see the small molecules. In our workflow, solvent presaturation is incorporated into two pulse sequences. The pulse sequences described below have been optimised using Bruker spectrometers (a system mastered by the authors). Nevertheless, these methods can be directly applied to other commercially available NMR systems, such as JEOL [22], including benchtop spectrometers [23]. In the first, on-resonance, continuous wave presaturation is employed during the relaxation delay to suppress water in a pulse-acquire experiment (zgpr). This sequence is employed to quantify the most abundant compounds, especially those that may be affected by suppression used in the presat-1D-NOESY-based sequence [24]. The second experiment suppresses water and ethanol using multiple frequency presaturation incorporated into the relaxation delay of a gradient-enhanced 1D-NOESY-based sequence (noesygpps1d) [11,24]. Suppression of water and ethanol results in a higher sensitivity for less abundant compounds. The gradient-enhanced 1D-NOESY-based sequence, in addition to several other benefits, affords a flat baseline; thus, it is often used in NMR metabolomic experiments [9,25]. A common additional experiment acquired in wine is the water-suppressed 2D ^1^H-^1^H J-resolved experiment [26]. Although the additional dispersion achieved through 2D experiments is helpful in resolving ambiguity, their use in automated workflows is still under development and is therefore not further discussed in this study.

#### 2.1.2. External Quantification Standard Development

Provided certain conditions are met, the intensity of the NMR signal is inherently proportional to the metabolite concentration, as it depends on the number of nuclei generating it and thus on the concentration of each metabolite. NMR quantification can be performed using an internal standard or an external standard with an analytical-grade reagent at a known concentration [27,28].

Trimethylsilylpropionate-2,2,3,3-*d*_4_ sodium salt (TMSP), commonly used to calibrate the chemical shift of spectra to 0.0 ppm, is sometimes also used as an internal quantification standard. However, it can be perturbed in some wines, making it unsuitable for use [13]. Other internal standards have been tested over the years. However, it is difficult to find one that remains stable in the matrix and is located in a neutral region of the spectrum (free from signal overlap). For instance, calcium formate (FCa) has shown promising results in wine analysis [9]. Nevertheless, the presence of formic acid in wines—even in trace amounts—will compromise the accuracy of the quantification.

**Figure 1:**
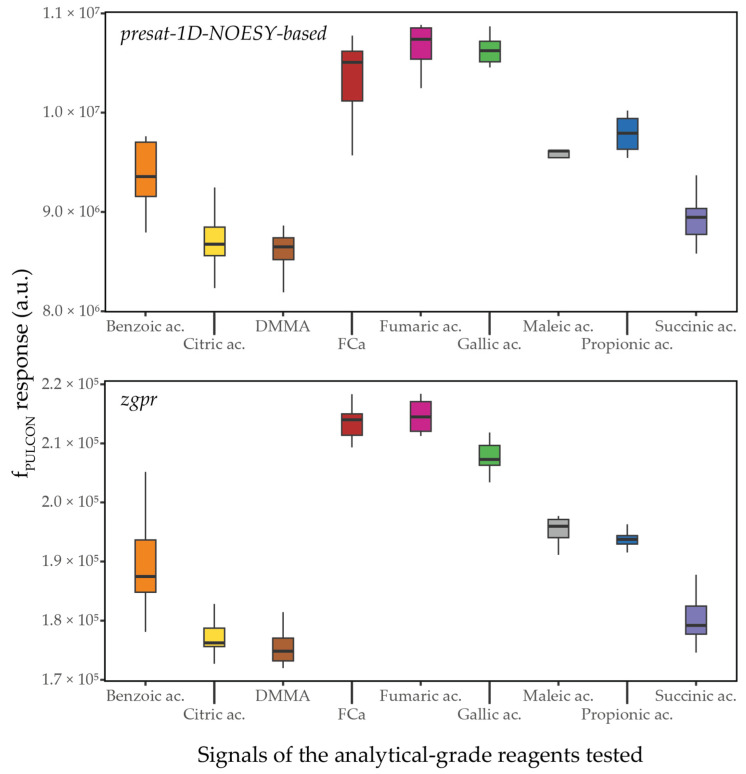
Response factors (*f*_PULCON_) for different standard compounds at various concentrations and in different mixtures, obtained from 1D-NOESY and *zgpr* experiments (FCa: Calcium formate; DMMA: dimethylmalonic acid; a.u.: arbitrary unit).

The stability of NMR instrumentation enables the use of external standards for quantitative analysis [27,28]. When spectra are acquired under the same conditions, an external standard can be used to calibrate the signal area for concentration determination. The main advantage of this approach lies in the absence of matrix interactions, which allows greater flexibility in the choice of the standard compounds. For this reason, we have adopted an external standard approach. Several candidate compounds were tested to select the most suitable standard solution composition (Figure 1). The pulse length-based concentration determination (PULCON) method was used to compare the standard compounds [12,29]. In theory, PULCON factors should remain consistent across different standard compounds obtained from analytical-grade reagents, as each is expected to yield a stable signal response regardless of concentration or matrix composition. Indeed, the calculation of this factor considers the concentration, the number of equivalent protons generating the signal, and the molar weight of the standard compound. In practice, when considered individually, each standard compound indeed shows a reproducible signal response in these conditions, with a coefficient of variation below 5% across all tested solution combinations with the standard compounds studied. Nevertheless, noticeable variations in the PULCON factor values are observed between standard compounds of different molecular nature, indicating that experimental conditions may influence the NMR response to some extent. Despite these differences, the high reproducibility observed for each individual standard compound supports the reliability of the external standard approach adopted in this study. To correct for any discrepancies and enhance the accuracy of quantification, correction factors (CFs) were calculated [12].

#### 2.1.3. Data Extraction from ^1^H-NMR Spectra

In this study, a total of 20 compounds were quantified, including eight organic acids (acetic, formic, fumaric, galacturonic, malic, shikimic, sorbic, and succinic acids), two sugars (α-glucose and β-glucose convert to total glucose concentration, and fructose), three alcohols (3-methylbutan-1-ol, glycerol, and methanol), four phenolic compounds (caffeic acid, catechin, epicatechin, and gallic acid), two esters (ethyl acetate and ethyl lactate), and an alkaloid (trigonelline). Signal assignments in the ^1^H-NMR spectrum were performed using our internal database [9] and spiking. The signals used for identification and quantification of the selected compounds are reported in Table 1.

To aid interpretation, dedicated spectral processing software was developed to extract quantitative data from wine 1D ^1^H-NMR spectral data. This open-source package, written in R, called RnmrQuant1D, is freely available online [30]. The package fully automates all processing steps—including Fourier transformation of the free induction decay (FID), phase and baseline correction, peak picking, peak fitting, integration of compound peak areas, calibration, and ultimately quantification of target compounds—thereby minimising operator-dependent biases.

**Figure 2:**
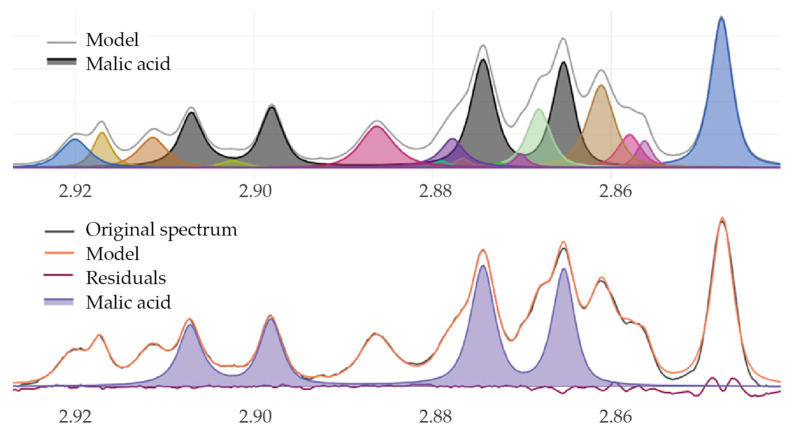
Malic acid peak annotation. **Top**: Identification of the malic acid signal pattern, peaks corresponding to the identified malic acid colored in grey within the region of interest of a red wine validation reference spectrum. Peaks in other colors represent the different peaks present in this region. **Bottom:** Overlay of the malic acid reference spectrum with the original and modelled spectra. Data were obtained using RnmrQuant1D 1.1.0 package.

For peak picking, a second derivative method was implemented to improve the spectrum fit, enabling better discrimination of moderately or strongly overlapping peaks [31]. For signal annotation, a quantification profile was defined for each compound [30], typically corresponding to a multiplet with minimal overlap in most wine samples. However, for some compounds, the peak detection pattern must be adjusted to reflect the content of a specific compound in wine more accurately. For example, by first principles, the malic acid signal at 2.89 ppm is a doublet of doublets with area of 1 proton, though computationally the peak is better detected by searching for two doublets of 0.5 proton intensity each at 2.871 and 2.903 ppm, both with *J* = 4.5 Hz (Table 1). Figure 2 illustrates the peak annotation corresponding to the identified malic acid pattern within the region of interest (ROI) of a wine spectrum (top) and shows the malic acid spectrum superimposed on both the original and modelled spectra (bottom).

### 2.2. Compound Quantification Method

#### 2.2.1. Correction Factor Determination

To ensure the intrinsic quantitative nature of NMR is maintained, a number of parameters need to be understood, optimised, and controlled [27]. For pulse sequences beyond the simple pulse-acquire experiment, effects such as relaxation result in losses, which means that these simple quantitative relationships are not held, requiring calibration to determine a correction factor (CF) to be used.

In our work, the CF was determined using the method of standard additions. The experimental design used for the method of standard addition complies with the OIV resolution OIV-OENO 418-2013. Direct spiking of standards into a wine reference sample ensures that the final matrix remains as close as possible to the initial wine sample, minimising matrix effects. This direct matrix spiking procedure enables the determination of a CF for each quantified compound to obtain its accurate concentration (see Section 3.6.3 and Equation (2)). Ideally, standard aliquots should be prepared directly in the wine reference solution. However, this approach requires large volumes of reference wine to achieve the necessary dilution steps across the calibration range, which complicates its practical use. To address this issue, an alternative strategy was adopted in this study: the model solution spiking. Standard aliquots were prepared in aqueous or hydroalcoholic solutions; aliquots were then added to the reference wine during sample preparation before sample pH adjustment. This model solution spiking approach is more practical, as it allows for the use of larger masses of analyte (reducing weight uncertainty) and simplifies the dilution process. To evaluate the robustness of this calibration method, three compounds were tested: sorbic acid, formic acid, and fumaric acid.

For formic acid, CFs of 0.74 (R^2^ = 0.999) and 0.73 (R^2^ = 0.999) were obtained using water and hydroalcoholic spiking solution, respectively. When direct spiking was performed into the wine reference solution, the CF was 0.84 (R^2^ = 0.999), resulting in an average absolute deviation of 0.10 CF units. For sorbic acid, CFs of 0.72 (R^2^ = 0.999), 0.85 (R^2^ = 0.998), and 0.77 (R^2^ = 0.998) for water, hydroalcoholic mixture, and direct spiking, respectively—corresponding to an average deviation of 0.06 CF units. For fumaric acid, CFs of 0.82 (R^2^ = 0.996), 0.90 (R^2^ = 0.994), and 0.92 (R^2^ = 0.995) were obtained, again yielding an average deviation of 0.06 CF units. These results suggest that matrix dilution influences the calibration curve and the resulting CF value. This observation is consistent with previous findings, such as those reported by Monakhova et al. [16]. However, the calibration procedures are specific to each laboratory and equipment setup. Moreover, CF values reported in prior NMR-based wine studies [12], including the OIV Type IV method (OIV-MA-AS316-01), suggest a standard uncertainty of approximately 10% (at a 95% confidence level), given that values are typically rounded to the nearest 0.05 CF units. Thus, the deviations observed in the present study remain within the acceptable limits of maximum allowable deviation (MAD) and were incorporated into the global uncertainty calculation. To confirm this approach, the measured concentrations—along with their associated uncertainties—were compared to the theoretical concentrations of the reference wine estimated using OIV official methods [4]. The theoretical concentrations fall within the experimental confidence interval (Figure S1). Nevertheless, for future calibration experiments, direct spiking into wine should be prioritised where feasible.

The CFs calculated for the 21 target signals are summarised in Table 2. The R^2^ ranged between 0.950 and 1.000, with most values exceeding 0.99, indicating a highly satisfactory performance of the calibration function. The lowest value was observed for caffeic acid (0.950), possibly due to various factors, including the low solubility of the standard, a low working concentration range, and the resulting complexity of the signal shape. An R^2^ of 0.988 for malic acid, while acceptable, may reflect spectral overlap in the region of interest, making signal deconvolution less accurate—particularly at low concentrations. The R^2^ of 0.983 for glucose (α + β) may be linked to that of β-glucose (R^2^ = 0.967), likely due to partial spectral overlap with tartaric acid. Similar explanations can be proposed for the R^2^ values of acetic acid (0.979), ethyl acetate (0.982), and ethyl lactate (0.955), possibly combined with minor effects of their chemical stability in aqueous solution.

#### 2.2.2. Accuracy of the Method: Linearity, Precision, and Trueness

The intrinsic linearity of ^1^H-NMR has been demonstrated across various studies [14,17,27]. In this work, this was further verified using formic acid through additional spiking during the calibration experiment. The spiking range extended from the typical concentration of formic acid in wine (approximately 10 mg/L) up to 25 g/L. A linear model fitted across this full range yielded an R^2^ > 0.999, confirming the high linearity of the method.

To assess precision, five series of acquisitions were performed for each wine sample used for validation. The intra-laboratory reproducibility standard deviation (S_RW_) was calculated and expressed as a percentage (u_prec_). The mean values are reported in Table 2. This standard uncertainty was then multiplied by a coverage factor of k = 2 corresponding to a 95% confidence interval, to obtain the expanded uncertainty expressed in percentage (U_prec_, or CV%). The obtained values were then compared with the MAD. For all validation materials, the results fall within the MAD-defined limits, indicating satisfactory precision (see Table S1).

Similarly, trueness was evaluated by calculating the relative bias between the measured and theoretical concentrations at each point in the validation set. The mean relative bias observed for each compound is presented in Table 2. For all compounds and validation points, the combined bias and standard precision uncertainty remained within MAD limits, confirming compliance with validation criteria (Table S1).

#### 2.2.3. Modelling Measurement Uncertainty and Limits of Quantification

**Figure 3:**
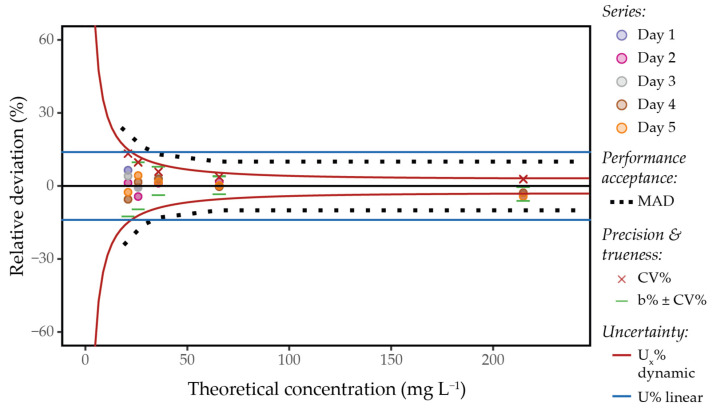
Accuracy profile of trigonelline determined from a validation set consisting of five reference materials (red wine), each analysed in triplicate across five independent series under intermediate precision conditions and quantified with RnmrQuant1D package over the gradient-enhanced 1D-NOESY-based NMR spectra. The mean relative deviation of each series is presented at each concentration level, along with the maximum allowable deviation (MAD) limits. Precision, trueness validation, and associated uncertainty models are also represented.

Measurement uncertainty was modelled as a linear function of concentration, integrating both bias and precision components, in accordance with OIV resolution OIV-OENO 418-2013. The parameters of the resulting linear uncertainty model, for a 95% confidence interval, are presented in Table 2 for each compound. However, this linear model has inherent limitations, particularly near the limits of quantification (LOQ), where analytical performance tends to degrade. As commonly observed, precision (expressed as CV%) tends to increase at lower concentrations—an effect clearly illustrated in the accuracy profile of trigonelline (Figure 3).

To address this behaviour, Thompson [18] proposed a dynamic uncertainty model that accounts for increased variability near the LOQ. This approach assumes that bias is negligible compared to the total uncertainty of the analytical method—an assumption that aligns with good practice recommendations in metrology. Indeed, the systematic bias of an analytical method is often identifiable and can then be either minimised or corrected. In practice, to model this uncertainty function, it is necessary to approach the method’s limits. Since NMR analysis is sensitive to matrix effects, wine must be used as reference material. Therefore, it is not always possible to approach the true LOQ owing to the intrinsic presence of certain non-target compounds in the wine matrix. However, dynamic modelling of uncertainty was used whenever possible.

For example, the validation set for caffeic acid includes concentration points close to the true LOQ, as the peak used for quantitation is typically free of non-target interference. As a result, the observed precision uncertainties for this compound are relatively high (Table S1), which affects the mean precision uncertainty estimation and directly impacts the final calculation of the global uncertainty, resulting in an overestimation. In comparison, the dynamic model reflects the uncertainty observed at low concentrations more accurately, where the precision is lower. It also fits well at higher concentrations, where uncertainty remains below 10%, thus better modelling the observed behaviour of the analytical method. The accuracy profiles of each compound are presented in Figure S2.

The accuracy profile of trigonelline is presented, as an example, in Figure 3 to illustrate the validation parameters assessed in the current work. Precision and bias components, represented as CV% and mean bias of measurement combined with precision uncertainty, remain within the MAD limits. Finally, it can be observed that dynamic uncertainty modelling provides a more accurate estimation of uncertainty at each concentration point compared to the linear model, which tends to overestimate uncertainty at higher concentrations and underestimate it near the LOQ.

According to OIV resolution OIV-OENO 418-2013, the limit of quantification (LOQ) is defined as the lowest amount of an analyte that can be quantitatively determined with an acceptable uncertainty. In the absence of normative or regulatory requirements, the acceptable uncertainty at the quantification limit is conventionally set to 60% of the LOQ. The LOQ estimates for each compound are provided in Table 2, based on the concentration at which the precision uncertainty (at 95% confidence interval) reaches 60%. When dynamic uncertainty modelling was not applicable, the signal-to-noise (S/N) ratio was used to estimate the LOQ, defined as the concentration corresponding to an S/N ratio of 10. This approach yielded values comparable to those obtained using the dynamic uncertainty model.

## 3. Materials and Methods

### 3.1. Chemicals, Solvents and Reagents

Dimethylmalonic acid (DMMA), maleic acid, calcium formate (FCa) of qNMR purity grade, and gallic acid were purchased from Supelco (Bellefonte, PA, USA). Succinic acid, citric acid monohydrate, propionic acid, benzoic acid, sorbic acid, caffeic acid, fumaric acid, and methanol were obtained from VWR Chemicals (Fontenay-sous-Bois, France). Glycerol, trigonelline, pyruvic acid, shikimic acid, L-malic acid, calcium formate (98%; used form spiking) were purchased from Acros Organics (Geel, Belgium) Sodium acetate, ethyl lactate, ethyl acetate, isoamyl alcohol (synonym for 3-methylbutan-1-ol), d-(+)-glucose, d-(–)-fructose, galacturonic acid sodium salt, (-)-epicatechin, and hydrated (+)-catechin were purchased from Sigma-Aldrich (St. Louis, MO, USA). All chemicals were of analytical or NMR grade.

Ultrapure water (H_2_O, Milli-Q system, Millipore, Molsheim, France) and absolute ethanol (EtOH, VWR Chemicals, Fontenay-sous-Bois, France) were used to prepare the standard solutions.

For the preparation of the deuterated buffer solution, deuterium oxide (D_2_O, 99.90% D) and 3-(trimethylsilyl)propionate-2,2,3,3-d_4_ sodium salt (TMSP, 98% D) were purchased from Eurisotop (Saint-Aubin, France), and phosphoric acid (85%), sodium azide (99.5%), and sodium dihydrogen phosphate (99%) were purchased from Sigma-Aldrich.

### 3.2. Deuterated Buffer and Internal Standard Solution

The deuterated buffer solution was prepared by Laboratoires Dubernet (Narbonne, France) according to a standardised protocol. Briefly, 1 M sodium dihydrogen phosphate and 1 M phosphoric acid solutions were prepared in D_2_O and mixed to obtain at least 100 mL of buffer at an apparent pH of 2.60. TMSP, added as an internal reference to calibrate the chemical shift to 0.0 ppm, was incorporated at a final concentration of 4.1 mM, along with sodium azide at 2.8 mM as an antimicrobial agent.

### 3.3. Quantification Standards

Prior to selecting the final composition of the external standards, various candidates were evaluated, including the set proposed by Teipel et al. [26]: benzoic acid, propionic acid, citric acid, succinic acid, gallic acid, maleic acid, fumaric acid, FCa, and DMMA. The compounds were dissolved in H_2_O and tested individually and in mixtures at different concentrations, with deuterated buffer solution added at a final proportion of 10% (*v*/*v*) in each solution. The resulting PULCON factor was calculated for each standard (see Section 3.6.3).

The final external quantification reference (QR) used for quantifications and the quality control solution (QC) used to ensure the reliability of the QR were prepared according to a standard operating procedure. Three stock solutions from analytical-grade reagents were prepared to approximate concentrations of 7 g/L for citric acid, 2.5 g/L for DMMA, and 5 g/L for succinic acid; exact concentrations were calculated from the weighted masses and purities of the standards. The QR was obtained by mixing 25 mL of the citric acid solution, 20 mL of the DMMA solution, and 5 mL of deuterated buffer solution. Similarly, 50 mL of QC was prepared using 20 mL citric acid, 20 mL DMMA, 5 mL succinic acid, and 5 mL deuterated buffer solution. Finally, 600 µL was transferred into a 5 mm NMR tube (maximum ± 1% inner diameter deviation, Standard Series Economy ST500-7 purchased from Eurisotop, Saint-Aubin, France; manufactured by Norell Inc., Morganton, NC, USA).

### 3.4. Samples

#### 3.4.1. Wines Used

The wine samples used in this study included Titrivin reference material AA5 (batch A 04201224 5) (Chambre d’Agriculture de la Gironde, Blanquefort, France) for calibration purposes. For validation experiments, a reference red wine was provided by Laboratoires Dubernet (Narbonne, France). These reference wines included a certificate of analysis that specified the initial concentrations of selected compounds, measured according to OIV-approved methods [4].

#### 3.4.2. Doping Solutions

Doping solutions were prepared to fortify the red wine references at known concentrations for targeted compounds to obtain calibration and validation sets. Analytical-grade reagents were used for all additions.

Two spiking strategies were compared to assess the robustness of correction factor determination in relation to matrix dilution effects for three compounds: sorbic, formic, and fumaric acids. For the model solution spiking, standard compounds were dissolved in either ultrapure water or a hydroalcoholic mixture (15% *v*/*v* ethanol in water), depending on solubility. Spiking solutions of varying concentrations were prepared by dilution in the corresponding solvent (i.e., water or hydroalcoholic mixture). For direct matrix spiking, standard compounds were added directly to the calibration red wine reference (Titrivin AA5); different concentrations were obtained by diluting the spiked wine with unspiked reference wine (Titrivin AA5). All dilutions were performed with class A volumetric glassware. Detailed compositions of the doping solutions are provided in Supplementary Material (Table S2).

For calibration and validation experiments involving the quantification of 20 compounds, spiking solutions were prepared according to the model solution spiking procedure (Tables S1 and S2). Calibration and validation sets were constructed using a five-point concentration range, with at least four replicates for calibration and three for validation.

#### 3.4.3. Sample Preparation

Wine samples (1.4 mL) were centrifuged at 14,000× *g* for 5 min at 15 °C. For the model solution spiking, 800 μL of wine supernatant was mixed with 100 μL of spiking solution and 100 μL of deuterated buffer. For the direct matrix spiking, 900 μL of wine supernatant at the desired concentration was mixed with 100 μL of deuterated buffer. In both cases, the pH was adjusted to 3.10 ± 0.02 using a semi-automatic small-scale system (BTpH, Bruker BioSpin, Rheinstetten, Germany) calibrated at pH 2 and 4, equipped with 1 M HCl and 1 M NaOH solutions. Finally, 600 μL of the prepared samples were transferred into 5 mm NMR tubes (Standard Series Economy ST500-7 purchased from Eurisotop, Saint-Aubin, France; manufactured by Norell Inc., Morganton, NC, USA) for NMR acquisitions.

### 3.5. ^1^H-NMR Data Acquisitions

The spectrometer used for ^1^H-NMR spectra acquisitions consisted of an Ascend 500 magnet with an Avance III 500 console (Bruker Biospin, Wissembourg, France) operating at 500.23 MHz under TopSpin (v 3.6.2). The system was equipped with a 5 mm BBI probe, automated tuning and matching accessory (ATMA), and an autosampler (Bruker Biospin). The temperature was set at 300 K using a chiller (BCU I) unit. Experiments were performed without sample spinning. Between each sample transfer, a 3 min equilibration period was applied for thermal stabilisation, followed by a temperature check before acquisition.

A modified zgpr pulse programme was used, and similarly for presat-1D-NOESY-based experiments, a modified noesygpps1d pulse programme was performed. To prevent sample heating, low-power, on-resonance water presaturation was applied during the last 15 s of the relaxation delay (d_1_). The mixing time (d_8_) was set to 10 ms. Multi-frequency presaturation was calculated using wavemaker to suppress signals from water (δ 4.80 ppm) and ethanol (δ 3.64 and δ 1.17 ppm). The power for the shaped presaturation pulse was set 6 dB higher than the power used for the zgpr presaturation. Pulsed field gradients with duration of 1 ms were used primarily to enforce the coherence transfer pathway and reduce spectral artefacts. The gradients were set to 50% and −10% for G_z_1 and G_z_2, respectively.

Before acquisition, an automation programme ensured all necessary instrument settings, including tuning, matching, locking, shimming, and calibration. Calibrations (the 90° hard pulse, centre frequency O_1_, and the presaturation pulse) were performed using the pulsecal, bt_calibrate_o1, and wavemaker functions. The obtained values were used for the two solvent-suppression sequences.

Acquisition parameters were as follows: relaxation delay (d_1_), 15 s; spectral width (SW), 16.0182 ppm; time domain (TD), 65,536 points; acquisition time (AQ), 4.0894 s; number of scans (NS) and dummy scans (DS), 4 and 2 for zgpr, and 64 and 8 for noesygpps1d; receiver gain (RG) was fixed, 4.5 for zgpr and 64 for noesygpps1d.

QR and QC samples were acquired under identical conditions as the wine samples, except for the relaxation delay, which was extended to 45 s to ensure complete signal relaxation for both pulse programmes. QR samples were acquired at the start of each series of acquisitions, and QC samples at least every 25 samples. All samples were acquired in triplicate. Each validation sample was acquired five times under intermediate precision conditions.

### 3.6. Automated Spectra Processing and Quantification

An R package named RnmrQuant1D has been developed to automate every step of the spectra processing [30]. Data were analysed with v 1.0.6 of this package under R version 4.4.3. To identify and then quantify the compounds, NMR spectra were first automatically processed to extract a list of peaks, which were then matched to patterns characteristic of the target compounds. These steps, described below, apply in the same way to the 1D ^1^H-NMR spectra acquired using the zgpr and the noesygpps1d pulse programme.

#### 3.6.1. ^1^H-NMR Spectra Preprocessing

The initial phase of spectra processing includes preprocessing, baseline correction, peak picking, and peak fitting.

The preprocessing step consists of Fourier transforming the FID after zero-filling once to reach a real spectrum size (SI) of 128 k. Exponential line broadening (LB) of 0.25 Hz is then applied, and the resulting spectrum is then phased automatically. To improve the zero-order phasing, the mean value around the TMSP signal is adjusted to be as close to zero as possible.

**Figure 4:**
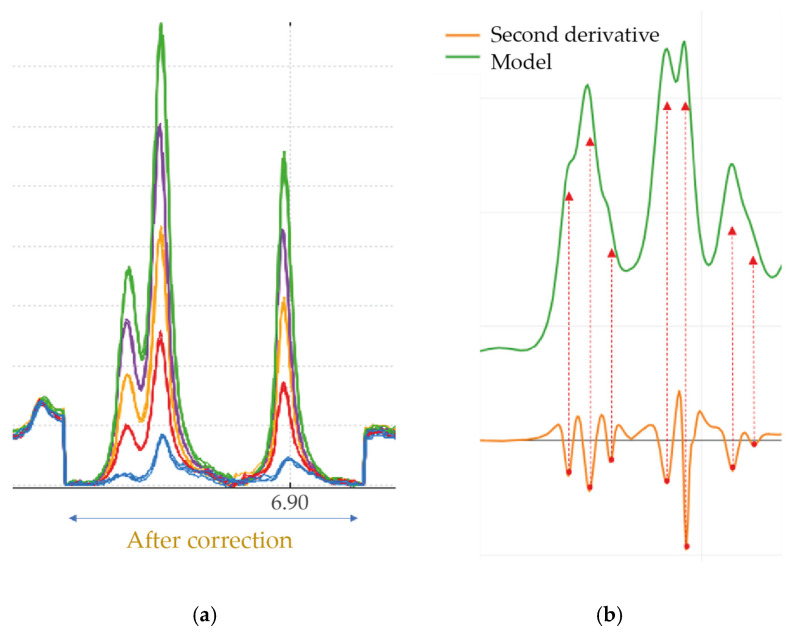
Preprocessing steps of a red wine ^1^H-NMR spectrum acquired using 1D-NOESY sequence. (**a**) Example of local baseline correction of several red wine NMR spectra in the 6.90 ppm region. (**b**) Peak detection on the original spectrum (green), obtained from its second derivative (orange) through negative intensity peaks.

Subsequent steps are applied only to spectral regions of interest (ROIs). Baseline correction (Figure 4a) is performed using the adaptive iteratively reweighted penalised least squares (airPLS) algorithm [32]. For optimal correction, the boundaries of each ROI are selected to be free of peaks. Peak picking in the ROIs is achieved using the second derivative method (Figure 4b). It should be noted, however, that the second derivative method is very sensitive to the signal-to-noise ratio, as differentiating the signal amplifies the noise level. Therefore, wavelet filtering (Daubechies and Symlets) is applied to the signal beforehand [33]. This filtering reduces noise without altering the signal’s shape. However, the method is no longer applicable for a signal-to-noise ratio below 3.

**Figure 5:**
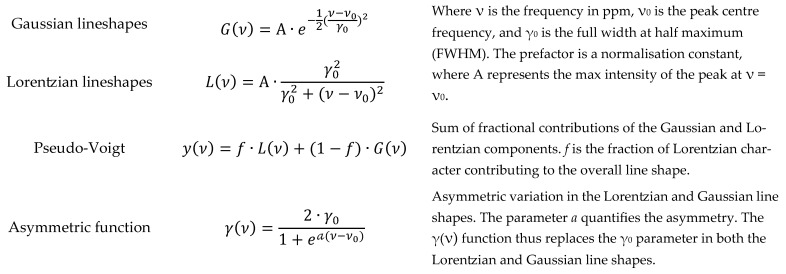
Gaussian and Lorentzian models combined into a pseudo-Voigt function, extended with asymmetric terms for peak fitting.

Finally, peak fitting is performed by modelling the spectrum curve within the ROIs. The selected model is a pseudo-Voigt function, defined as the weighted sum of a Lorentzian and a Gaussian (Figure 5). A parameter is introduced to adjust the Lorentzian contribution, where the Gaussian contribution is determined as its complement to 1. In addition, peaks in certain zones affected by pulse sequences involving one or more presaturation steps could display asymmetry. To account for this, an additional parameter is introduced into the model to consider a possible peak asymmetry [34]. These pseudo-Voigt curves are fitted to the data points using a simple least-squares cost function, optimised using the gradient method. During the peak fitting step, a quality control procedure is applied to each spectrum, determining the full width at half maximum (FWHM) of the internal standard peak (TMSP). The spectrum quality is accepted when FWHM of the TMSP peak is below 1 Hz [35].

#### 3.6.2. Area Extraction of Targeted Compounds

**Figure 6:**
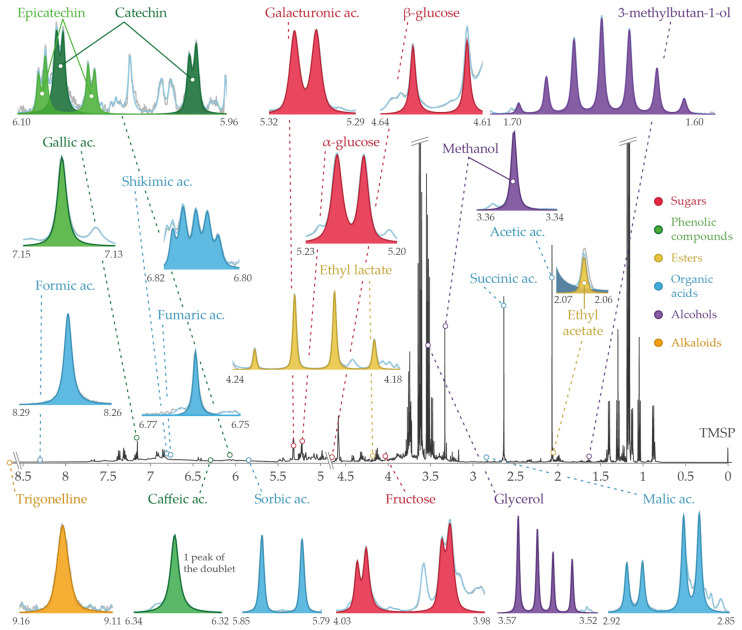
Assignments based on fully automated ^1^H-NMR spectra processing, including signal deconvolution, performed using RnmrQuant1D for the 21 targeted signals corresponding to the 20 selected compounds.

Once the peak list is obtained, the next phase involves searching for the pattern of the targeted compounds within the peak list of the ROIs. This process relies on a list of compound-specific patterns (Table 1) established from previous spiking experiments and a laboratory database. A file referred to as the “quantification profile” defines the set of signatures for each compound in the relevant ROIs [30]. This profile enables the configuration of all steps in spectrum processing, from preprocessing to peak attribution and integration, and finally to the quantification of the targeted compounds in the wine sample. These settings were optimised on a set of commercial red wines previously analysed in the laboratory to ensure accurate pattern recognition of targeted compounds, in accordance with identification reported in Table 1, as illustrated in Figure 6.

Due to the potential chemical shift variations between the spectra of the same compound, it is possible to calibrate the ROI using a peak within or outside this region to facilitate the search for the targeted patterns. To this end, both the ppm range and the central value of the peak to be calibrated can be specified. Alternatively, each spectrum can be recalibrated prior to any processing by specifying the calibration peak in the preprocessing section of the quantification profile. In this study, the central peak of the ^13^C satellite of ethanol triplet was calibrated at 1.046 ppm. Because ppm calibration is applied across the entire spectrum, each ppm value specified in the other sections of the quantification profile is automatically adjusted to this new scale. For further details, please refer to the online documentation of the R package RnmrQuant1D 1.1.0 [30].

#### 3.6.3. Quantification Using a PULCON-Based Approach

Analyte concentrations are determined from the integrals of their characteristic resonances using the pulse length-based concentration determination (PULCON) method [36,37]. This well-established approach employs an external standard of known concentration to assess the NMR spectrometer response to a defined number of resonating nuclei under specific acquisition conditions. The resulting normalised response, known as the PULCON factor (*f*_PULCON_), is calculated according to the equation shown below:

(1) $$f_{PULCON}=I_{ref}.\frac{K\cdot {MW}_{ref}}{{NH}_{ref}\cdot {MC}_{ref}},\;\mathrm{with}\;K=\frac{{SW}_{ref}}{{SI}_{ref}},$$

$I_{ref}$ is the absolute integral of a selected resonance of the standard, ${MW}_{ref}$ corresponds to its molecular weight, ${NH}_{ref}$ to the number of protons contributing to selected resonance, ${MC}_{ref}$ to the exact mass concentration of the standard, ${SW}_{ref}$ denotes the spectral width, ${SI}_{ref}$ the size of the real spectrum.

A dedicated file called calibration profile (Table 3) is used to calibrate the quantification parameters based on external standard spectra. This profile enables full automation of the calibration procedure. Further details can be found in the online documentation of the RnmrQuant1D package [30].

To obtain insights into the quantitative composition of samples, the PULCON factor is first automatically calculated for the QR sample. The relative difference between the *f*_PULCON_s determined for citric acid and DMMA must remain below 5%.

In compliance with the international standard ISO 17025 [38], which requires verification and confirmation of standard-based quantification using an independently prepared control solution, the QC sample is assessed for precision. For each series of measurements, the concentration of the QC sample standards is also automatically calculated. These values had to remain within ± 8% of their initial values at the time of preparation.

Finally, the PULCON factor is applied to calculate the concentration of each selected compound in the wine samples by the following formula:

(2) $$C_{x}=\frac{I_{x}\cdot {SW}_{x}\cdot {MW}_{x}\cdot {NS}_{ref}\cdot {P1}_{x}}{{SI}_{x}\cdot f_{PULCON}\cdot {NH}_{x}\cdot {NS}_{x}\cdot f_{x}^{dil}\cdot {P1}_{ref}}\cdot CF,$$

where the notations are the same as those for *f*_PULCON_. C_x_ is the mass concentration of the selected compound, *NS_x_* and *NS_ref_* are the numbers of scans for the two experiments, *P*1*_x_* and *P*1*_ref_* are the 90° pulse lengths, *CF* is the correction factor, and $f_{x}^{dil}$ is the sample dilution factor.

### 3.7. Evaluation of Validation Parameters

#### 3.7.1. Calibration Steps and Calculation of Correction Factor

Quantification of the compounds was performed after calibration in accordance with OIV resolution OIV-OENO 418-2013. The correction factor (CF) of each compound was determined using a five-point calibration set (see Section 3.4). A linear regression model was built to predict the spiked concentrations from the measured ones. The slopes of the regression lines provided the CFs.

#### 3.7.2. Calculation and Model Used for the Assessment of Validation Parameters

Evaluation of validation parameters (trueness, precision, uncertainty, and limits of quantification) was carried out according to the resolution OIV-OENO 418-2013. The maximum allowable deviation (MAD) of the analytical method relative to accepted reference values was established for each validation sample at the different concentrations tested (Table S1). The MAD values were set to ensure compliance with analytical performance requirements, considering calibration, precision, and trueness.

The standard measurement uncertainty u(x) was modelled as a linear function of concentration and expanded by a factor of k = 2 ($U_{k=2}$), corresponding to a 95% confidence interval. All uncertainties were expressed as concentration percentages, unless otherwise specified. It was based on the combination of systematic error (bias) and random error (linked to measurement precision), calculated as follows:

(3) $$u\left(x\right)=\sqrt{{u_{bias}}^{2}+{u_{prec}}^{2}},\;\mathrm{and}\;U_{k=2}=\pm 2\times u(x).$$

The precision component, $u_{prec}$, was estimated from the validation set under intermediate precision conditions. It corresponds to the intra-laboratory reproducibility standard deviation ($S_{RW}$), then expressed in percentage, calculated as:

(4) $$S_{RW}=\sqrt{{s_{\bar{x}}}^{2}+\left(1-\frac{1}{p}\right)\times {s_{r}}^{2}},\;\mathrm{and}\;{CV\%}_{k=2}=\pm 2\times \frac{S_{RW}}{x},$$

where $s_{\bar{x}}$ is the standard deviation of within-run averages, *p* is the number of replicates, and $s_{r}$ is the repeatability standard deviation (calculated as the mean of within-run variances). The bias component (in %), $u_{bias\;}$, was calculated with the following formula:

(5) $$u_{bias}=\sqrt{{u_{MTD}}^{2}+\frac{\sum_{i=1}^{p}{u_{{ref}_{i}}}^{2}}{p}},$$

where $u_{MTD}$ (%) is the standard uncertainty of the maximum tolerated deviation, here assumed equivalent to the stabilised MAD, and $u_{{ref}_{i}}$ (%) is the uncertainty arising from preparation steps for each reference material of the validation set, including the uncertainty of the initial reference value (from the certificate of analysis based on OIV-approved methods). The latter contribution was negligible compared to that of $u_{MTD}$.

As an alternative to the standard approach, when possible, uncertainty was modelled according to Thompson’s method [18]. This approach assumes that the systematic error is negligible or corrected. For each point of the validation range, ${CV\%}_{k=2}$ was calculated as in Equation 4. At low concentrations close to limit of quantification, ${CV\%}_{k=2}$ increases. The parameter α was calculated from two points located at the inflection of this increase using Equation 6. While the parameter β was determined at higher concentrations where ${CV\%}_{k=2}$ stabilises (Figure 3). Uncertainty $U_{x}\;$(%) as a function of concentration is then expressed as:

(6) $$\alpha =x1\cdot x2\times \sqrt{\frac{{y_{1}}^{2}-{y_{2}}^{2}}{{x_{2}}^{2}-{x_{1}}^{2}}},\;\mathrm{giving}\;U_{x}=\sqrt{\frac{\alpha^{2}}{x^{2}}+\beta^{2}}.$$

The trueness performance was verified by evaluating the relative bias (b in %) between measured and theoretical concentrations of the validation set. It must comply with the following inequalities:

(7) $$+MAD>b+{CV\%}_{k=2}\;\mathrm{and}\;-MAD<b-{CV\%}_{k=2}.$$

Finally, limits of quantification (LOQ) were determined using the uncertainty model, with LOQ defined as the concentration at which $U_{x}$ reaches approximately 60% (OIV resolution OIV-OENO 418-2013). When this approach was not applicable, an alternative estimation was obtained using signal-to-noise ratio (S/N), following the procedure described by Monakhova et al. [16], where LOQ corresponds to an S/N of 10. Since spiking was performed at known concentrations, the LOQ could be estimated directly from the S/N observed at these levels.

## 4. Conclusions

^1^H-NMR-based metabolomics is a highly valuable approach for ensuring wine traceability, including in official regulatory contexts, as it allows the quantification of a diverse range of wine compounds in a single experiment. While an initial NMR method has been accepted by OIV, it remains limited to only six compounds (Type IV method OIV-MA-AS316-01) and relies on spectral processing tools that are not open-source. In this work, we have developed a standardised, open-source NMR measurement and data analysis workflow to overcome these limitations.

We present a fully validated quantitative ^1^H-NMR method for 20 oenologically relevant compounds, integrating open-source standard operating procedures and automated data processing. The method enables rapid, accurate quantification of diverse wine constituents—including organic acids, sugars, esters, alcohols, phenolics, and an alkaloid—with minimal sample preparation and consumption and efficient analysis times. Importantly, it provides absolute concentrations, facilitating integration into harmonised databases and enabling comparability across laboratories and analytical platforms.

Beyond rigorous technical validation, the workflow presents significant operational advantages: it standardises analyses, supports future expansion to additional compounds, and can potentially be implemented on benchtop NMR spectrometers, making NMR-based wine analysis more accessible. The development of a dedicated graphical user interface will further streamline data extraction, making the workflow accessible to non-expert users.

Altogether, this standardised ^1^H-NMR quantification protocol combines versatility, speed, and reproducibility, establishing a robust, transferable, and practical tool for wine authentication, quality control, and future integrative approaches in oenological research. By bridging methodological rigour with practical applicability, this work lays the foundation for broader adoption of NMR in regulatory and research settings, thereby enhancing the traceability and authenticity of high-value wines.

## Figures and Tables

**Table 1:** Assignments of the signals for each targeted compound, including chemical shift, multiplicity, coupling constant, and corresponding protons.

Compound	ChEBI ID	δ (ppm)	Multiplicity ^3^	*J* (Hz)	Proton Moiety
3-Methylbutan-1-ol	15837	1.65	m	-	CH
Acetic acid	15366	2.08	s	-	CH_3_
α-Glucose	17925	5.23	d	3.6	CH
β-Glucose	15903	4.63	d	7.9	CH
Caffeic acid	16433	6.33	d	16	CH
Catechin ^1^	23053	5.99 and 6.09	d	2.0	CH
Epicatechin ^1^	90	6.07 and 6.10	d	2.0	CH
Ethyl acetate	27750	2.07	s	-	CH_3_
Ethyl lactate	78321	4.21	q	7.1	CH_2_
Formic acid	30751	8.27	s	-	CH
Fructose	28757	4.01	dd	12.8; 1.2	CH_2_
Fumaric acid	18012	6.75	s	-	2CH
Galacturonic acid	33830	5.30	d	3.7	CH
Gallic acid	30778	7.16	s	-	2CH
Malic acid	6650	2.89	dd	16.3; 4.5	CH
Shikimic acid	16119	6.82	m	-	CH
Sorbic acid	35962	5.82	d	15.3	CH
Succinic acid	15741	2.65	s	-	2CH_2_
Trigonelline	229203	9.14	s	-	CH
Glycerol ^2^	17754	3.55	dd	11.8; 6.5	2CH
Methanol ^2^	17790	3.35	s	-	CH_3_

^1^ Signals of different proton groups are used. ^2^ Quantification is performed with water suppression (*zgpr*) NMR spectra. ^3^ Abbreviations: s = singlet; d = doublet; dd = doublet of doublets; q = quartet; m = multiplet.

**Table 2:** Overview of correction factors (CF) for red wine samples and validation parameters for each selected compound, comprising calibration fit, associated uncertainties, and quantification limits. Notations: µ(b) = mean of bias observed in validation experiments (%); µ(u_prec_) = mean of u_prec_ observed at each point of concentration in the validation set expressed (%); u_bias_ = bias uncertainty related to each compound (%); U_k=2_ = linear uncertainty (%); α and β = parameters of dynamic uncertainty model.

Compound	CF	R^2^	µ(b)	µ(u_prec_)	u_bias_	U_k=2_	A	β	LOQ
3-Methylbutan-1-ol	0.75	0.998	−15.86	3.42	15.30	31.36	-	-	10 ^b^
Acetic acid	0.55	0.979	−23.10	2.25	20.22	40.69	-	-	1 ^b^
Glucose (based on α-glucose signal)	2.30	0.992	5.03	4.40	10.44	22.66	-	-	40 ^b^
Glucose (based on β-glucose signal)	1.25	0.967	−0.27	3.57	10.44	22.07	-	-	15 ^b^
Glucose (α + β)	0.80	0.983	−0.35	3.47	12.85	26.62	-	-	-
Caffeic acid	0.80	0.950	−19.68	18.93	10.44	43.24	6.5039	0.0757	10 ^a^
Catechin	0.90	0.999	2.13	4.34	8.08	18.34	8.5548	0.0430	15 ^a^
Epicatechin	0.75	0.998	−6.83	9.99	10.44	28.90	11.5328	0.0873	20 ^a^
Ethyl acetate	0.95	0.982	−31.19	13.86	30.15	66.37	24.9662	0.1991	45 ^a^
Ethyl lactate	0.90	0.955	−9.06	11.60	22.70	50.98	-	-	5 ^b^
Formic acid	0.75	1.000	−4.19	5.93	10.44	24.01	0.6999	0.0465	1 ^a^
Fructose	2.20	0.997	−3.91	2.36	10.44	21.41	-	-	20 ^b^
Fumaric acid	0.80	0.997	8.37	6.99	10.44	25.13	1.4600	0.0800	5 ^a^
Galacturonic acid	1.95	0.998	−2.47	1.62	8.08	16.48	-	-	50 ^b^
Gallic acid	0.75	0.994	−10.24	6.83	17.76	38.06	-	-	1 ^b^
Malic acid	1.10	0.988	−0.19	8.86	20.22	44.15	5.8189	0.1091	10 ^a^
Shikimic acid	0.75	0.999	6.98	4.63	5.83	14.89	4.6924	0.0419	10 ^a^
Sorbic acid	0.90	0.999	8.08	6.69	10.44	24.80	5.9122	0.0902	10 ^a^
Succinic acid	1.05	0.955	3.25	4.67	15.30	31.99	-	-	1 ^b^
Trigonelline	0.90	0.975	−0.03	3.80	5.83	13.92	3.0739	0.0280	5 ^a^
Glycerol	1.25	0.990	39.27	2.46	25.18	50.60	-	-	1 ^b^
Methanol	0.60	0.997	−34.55	2.58	25.18	50.62	-	-	15 ^b^

^a^ LOQ in mg/L calculated for an uncertainty of 60% based on dynamic modelled uncertainty. ^b^ LOQ in mg/L determined for signals with signal-to-noise ratio (S/N) of 10.

**Table 3:** Calibration table for external standards (QC: control quality sample; QR: quantification standard reference sample; MW: molecular weight; NH: number of protons; MC: mass concentration in mg/L; ppm1 and ppm2: spectral range used for deconvolution).

Sample	Compound	MW	NH	MC	ppm1	ppm2
QC	DMMA	132.11	6	999.437	1.300	1.500
QC	Succinate	118.09	4	501.395	2.600	2.735
QC	Citrate1	210.14	2	2811.870	2.735	2.910
QC	Citrate2	210.14	2	2811.870	2.910	3.100
QR	DMMA	132.11	6	999.437	1.300	1.500
QR	Citrate1	210.14	2	3514.838	2.735	2.910
QR	Citrate2	210.14	2	3514.838	2.910	3.100

## Data Availability

All data generated or analysed during this study are available from the corresponding author on reasonable request.

## Supplementary Materials

The following supporting information can be downloaded at https://www.mdpi.com/article/10.3390/molecules31010065/s1, Table S1: Theoretical and experimental concentrations (in mg/L) and validation parameters for each reference material, Table S2: Concentrations of spiked standards used for calibration, Figure S1: Relative deviation (%) between measured and theoretical values in the reference wine prepared with a 90:10 *v*/*v* mixture of wine and deuterated buffer solution, Figure S2: Accuracy profiles for all compounds obtained using the validation set.

## Abbreviations

The following abbreviations are used in this manuscript:

CF	Correction factor
DMMA	Dimethylmalonic acid
FCa	Calcium formate
FID	Free induction decay
FWHM	Full width at half maximum
MAD	Maximum allowable deviation
OIV	International Organisation of Vine and Wine
PULCON	Pulse Length-Based Concentration Determination
QC	Quality control standard
QR	Quantification standard reference
ROI	Region of interest
TMSP	3 (trimethylsilyl)propionate 2,2,3,3 d4 sodium salt
