# Supplementary material for: 1H-NMR Analysis of Wine Metabolites: Method Development and Validation
Source: Molecules. 2025 Dec 24;31(1):65. doi: 10.3390/molecules31010065 (PMC12787021; doi:10.3390/molecules31010065)
Supplement: Supplementary file 1 [file molecules-31-00065-s001.zip › molecules-4010360-supplementary.pdf]

molecules

MDPI

Supplementary material

^{1}H-NMR analysis of wine metabolites: Method development and validation

Guillaume Leleu [1], Rémi Butelle [1], Daniel Jacob [2,3,†], Lou-Ann Kurkiewicz [1], Jean-Claude Boulet [4], Catherine Deborde [2,3,†], Matthieu Dubernet [5], Laetitia Gaillard [6], Antoine Galvan [6], Karen Gaudin [1], Alexandra Gossé [7], Markus Herderich [8,9,10], Annick Moing [2], Sophie Rosset [6], Flynn Watson [9,10], Gregory Da Costa [1] and Tristan Richard [1,*]

[1] Univ. Bordeaux, Bordeaux INP, INRAE, OENO, UMR 1366, ISVV, F-33140 Villenave d'Ornon, France; guillaume.leleu@u-bordeaux.fr (G.L.); remi.butelle@u-bordeaux.fr (R.B.); lou-ann.kurkiewicz@u-bordeaux.fr (L.-A.K.); karen.gaudin@u-bordeaux.fr (K.G.); gregory.da-costa@u-bordeaux.fr (G.D.C.)
[2] INRAE, Univ. Bordeaux, Biologie du Fruit et Pathologie, UMR 1332, Centre INRAE de Nouvelle Aquitaine Bordeaux, F-33140 Villenave d'Ornon, France; daniel.jacob@inrae.fr (D.J.); catherine.deborde@inrae.fr (C.D.); annick.moing@inrae.fr (A.M.)
[3] INRAE, CALIS/PROBE Research Infrastructures, BIBS Facility, F-44300 Nantes, France
[4] INRAE, Institut Agro Montpellier, Univ. Montpellier, SPO, UMR 1083, F-34060 Montpellier, France; jean-claude.boulet@inrae.fr
[5] Laboratoire Dubernet, 35 Rue de Combe du Meunier, 11100 Montredon-des-Corbières, France; matthieu.dubernet@dubernet.com
[6] Service Commun des Laboratoires, 3 Avenue du Dr. Albert Schweitzer, F-33600 Pessac, France; laetitia.gaillard@scl.finances.gouv.fr (L.G.); antoine.galvan@scl.finances.gouv.fr (A.G.); sophie.rosset@scl.finances.gouv.fr (S.R.)
[7] Les Grands Chais de France, 1 Rue de la Division Leclerc, F-67290 Petersbach, France; alexandra.gosse@lgcf.fr
[8] The University of Adelaide, Waite Research Institute, Urrbrae, SA 5064, Australia; markus.herderich@awri.com.au
[9] The Australian Wine Research Institute, P.O. Box 46, Glenside, SA 5065, Australia; flynn.watson@awri.com.au
[10] Metabolomics Australia, P.O. Box 46, Glenside, SA 5065, Australia
* Correspondence: tristan.richard@u-bordeaux.fr
† Current address: INRAE, UR 1268 Biopolymères Interactions Assemblages, F-44300 Nantes, France.

 https://doi.org/10.3390/molecules31010065

Table S1. Theoretical and experimental concentrations (in mg/L), and validation parameters for each reference material (b: mean bias of all measurements of the five series expressed in %; s_r: repeatability standard deviation; s_x: standard deviation of within-run averages; S_{RW}: intra-laboratory reproducibility standard deviation; u_{prec}: S_{RW} expressed as a percentage of the measured concentration; and MAD: maximum admissible deviation in %.

Compounds	Concentration		b	s_r	s_x	S_{RW}	u_{prec}	MAD
	Theo [a]	Exp. [b]						
3-methylbutan-1-ol	221.50	173.94	-21.47	4.40	2.31	4.27	2.45	30
	251.55	216.31	-14.01	7.25	4.5	7.44	3.44	30
	259.06	209.81	-19.01	11.46	2.43	9.67	4.61	30
	264.42	219.87	-16.85	8.03	5.53	8.58	3.9	30
	281.59	238.99	-15.13	8.77	5.25	8.88	3.72	30
	296.62	252.13	-15.00	7.15	5.45	7.99	3.17	30
	321.66	272.93	-15.15	7.08	3.68	6.85	2.51	30
	521.97	468.94	-10.16	15.20	10.57	16.3	3.48	30
Acetic acid	380.00	303.05	-20.25	5.80	6.09	7.71	2.54	40
	384.37	304.61	-20.75	4.53	2.17	4.29	1.41	40
	388.74	307.53	-20.89	8.37	5.1	8.53	2.77	40
	392.86	311.14	-20.80	7.60	2.6	6.73	2.16	40
	401.86	307.02	-23.60	4.33	2.01	4.07	1.33	40
	411.23	315.86	-23.19	6.60	3.59	6.48	2.05	40
	598.58	405.66	-32.23	7.00	13.05	14.25	3.51	40
Caffeic acid	10.60	6.51	-38.59	1.99	1.2	2.02	31.03	200
	15.51	11.71	-24.52	3.05	1.44	2.88	24.59	110
	20.43	15.68	-23.25	3.93	1.82	3.69	23.54	105
	30.26	24.91	-17.69	3.34	1.03	2.92	11.72	50
	207.19	218.89	5.65	8.69	4.29	8.29	3.79	20
Catechin	53.30	55.19	3.55	1.88	0.67	1.67	3.03	25
	58.18	58.70	0.91	3.66	2.63	3.98	6.78	22
	63.05	63.66	0.96	3.22	1.31	2.94	4.62	20
	72.80	73.74	1.29	3.48	2.51	3.79	5.14	17
	248.30	258.08	3.94	6.58	1.39	5.55	2.15	15
Epicatechin	30.90	27.24	-11.83	2.96	1.32	2.75	10.09	55
	35.40	32.33	-8.67	3.89	3.8	4.95	15.31	50
	39.90	36.98	-7.33	3.87	1.76	3.62	9.79	40
	48.91	45.59	-6.78	3.86	3.54	4.74	10.4	35
	211.00	211.95	0.45	8.21	6.37	9.25	4.36	20
Ethly lactate	63.60	56.88	-10.57	5.90	3.05	5.7	10.02	45
	161.29	145.07	-10.06	18.53	8.85	17.53	12.08	45
	185.72	164.32	-11.52	20.91	5.41	17.91	10.9	45
	203.16	195.56	-3.74	22.41	7.07	19.62	10.03	45
	258.99	241.58	-6.72	42.66	8.89	35.95	14.88	45
	307.83	264.80	-13.98	32.58	28.2	38.77	14.64	45
	389.24	379.24	-2.57	34.13	21.86	35.42	9.34	45
	1040.53	902.87	-13.23	84.94	70.47	98.87	10.95	45
Ethyl acetate	60.40	36.35	-39.82	4.98	4.93	6.39	17.58	80
	72.52	50.61	-30.22	8.39	3.35	7.63	15.08	60
	78.23	55.19	-29.45	7.31	2.45	6.45	11.69	60
	90.71	69.36	-23.53	9.35	5.46	9.39	13.54	60
	103.70	74.45	-28.20	12.60	4.99	11.43	15.35	60
	363.48	232.84	-35.94	27.35	6.22	23.18	9.95	60
Formic acid	0.00	0.00	0.00	0.23	0.28	0.34	20.89	80
	4.15	4.11	-0.82	0.22	0.08	0.2	3.49	60

	8.29	8.21	-1.02	0.36	0.28	0.41	4.18	40
	12.19	12.36	1.36	0.42	0.14	0.37	2.64	30
	20.73	18.78	-9.42	0.59	0.17	0.51	2.52	20
	29.62	27.42	-7.42	1.64	0.84	1.58	5.46	20
	207.31	182.37	-12.03	3.74	2.98	4.27	2.32	20
Fructose	1150.00	1087.90	-5.40	22.61	13.88	23.1	2.12	20
	1249.17	1181.72	-5.40	32.68	32.63	42.15	3.57	20
	1273.97	1210.14	-5.01	31.04	21.21	33.05	2.73	20
	1291.68	1230.45	-4.74	27.00	9.6	24.04	1.95	20
	1348.35	1304.26	-3.27	25.06	19.03	27.94	2.14	20
	1397.94	1336.57	-4.39	21.71	9.93	20.32	1.52	20
	1480.58	1429.94	-3.42	18.44	12.31	19.45	1.36	20
	2141.74	2150.10	0.39	66.31	48.04	72.38	3.37	20
Fumaric acid	4.88	6.26	28.27	0.49	0.23	0.46	7.35	60
	9.76	9.88	1.21	1.14	0.16	0.94	9.52	30
	19.52	19.52	0.01	1.11	1.03	1.37	7.02	20
	195.17	202.97	4.00	7.38	5.72	8.31	4.09	20
Galacturonic acid	592.42	592.42	0.00	7.69	3.56	7.22	1.22	15
	609.51	592.02	-2.87	6.96	5.03	7.59	1.28	15
	626.61	622.72	-0.62	6.51	4.88	7.22	1.16	15
	642.69	641.34	-0.21	7.79	5.63	8.49	1.32	15
	677.88	647.04	-4.55	13.80	7.06	13.3	2.06	15
	714.51	692.93	-3.02	14.20	2.8	11.93	1.72	15
	1447.03	1359.78	-6.03	35.15	20.71	35.39	2.6	15
Gallic acid	32.50	27.85	-14.30	1.93	1.97	2.52	9.05	35
	38.48	38.45	-0.07	2.91	1.35	2.73	7.1	35
	44.45	39.34	-11.51	1.26	1.28	1.64	4.17	35
	50.08	44.37	-11.39	4.22	1.98	3.97	8.95	35
	62.38	52.13	-16.44	3.82	2.38	3.92	7.52	35
	75.19	68.45	-8.97	2.76	2.16	3.12	4.56	35
	331.33	301.45	-9.02	16.17	14.33	19.48	6.46	35
Glycerol	7480.00	10709.86	43.18	358.09	104.4	310.5	2.9	50
	7499.87	10361.07	38.15	204.80	132.8	213.5	2.06	50
	7519.74	10499.82	39.63	274.12	131.6	259.7	2.47	50
	7538.45	10635.99	41.09	255.62	45.96	213.7	2.01	50
	7579.36	10364.01	36.74	398.41	122.7	347.7	3.35	50
	7621.94	10429.86	36.84	166.37	155.5	206.5	1.98	50
Malic acid	17.42	14.95	-14.21	1.54	1.77	2.17	14.52	60
	20.85	20.47	-1.81	1.82	1.2	1.91	9.33	50
	27.70	24.82	-10.38	1.58	0.97	1.61	6.49	45
	150.96	188.26	24.71	10.38	5.8	10.27	5.46	40
Methanol	134.10	81.75	-39.04	1.69	0.83	1.61	1.97	50
	155.54	97.94	-37.03	2.00	1.68	2.34	2.39	50
	176.97	114.27	-35.43	2.21	0.58	1.9	1.66	50
	197.15	130.71	-33.70	2.00	1.14	1.99	1.52	50
	241.29	162.27	-32.75	5.77	4.32	6.39	3.94	50
	287.22	202.98	-29.33	7.94	4.96	8.16	4.02	50
Shikimic acid	28.80	31.03	7.73	1.80	1.83	2.35	7.57	50
	40.62	43.94	8.18	3.12	1.15	2.8	6.37	35
	46.18	49.81	7.86	3.27	1.35	2.99	6	30
	58.35	60.79	4.18	1.84	0.62	1.63	2.68	25
	71.01	78.71	10.84	2.50	1.27	2.4	3.05	20

	324.28	334.39	3.12	6.81	4.25	7	2.09	10
Sorbic acid	9.94	13.62	37.00	1.75	0.38	1.48	10.87	65
	19.88	22.21	11.72	1.50	1.19	1.71	7.7	35
	49.70	45.95	-7.55	1.44	1.22	1.69	3.68	20
	198.80	181.21	-8.85	5.23	6.97	8.17	4.51	20
Succinic acid	800.00	790.00	-1.25	23.42	29.3	34.99	4.43	30
	899.23	916.68	1.94	26.23	24.67	32.67	3.56	30
	924.04	941.23	1.86	29.83	29.66	38.38	4.08	30
	941.76	963.42	2.30	45.66	35.34	51.37	5.33	30
	998.47	1029.02	3.06	39.93	26.29	41.88	4.07	30
	1048.09	1085.08	3.53	47.08	17.22	42.12	3.88	30
	1792.34	1995.95	11.36	133.04	95.84	144.9	7.26	30
Total glucose (α + β)	1190.00	1227.96	3.19	17.80	22.42	26.72	2.18	25
	1289.67	1298.31	0.67	16.95	27.9	31.14	2.4	25
	1314.59	1318.80	0.32	52.89	15.3	45.81	3.47	25
	1332.39	1351.57	1.44	37.85	16.55	35.06	2.59	25
	1389.34	1393.23	0.28	33.68	20.12	34.07	2.45	25
	1439.18	1406.65	-2.26	144.70	72.92	138.8	9.87	25
	1522.24	1369.40	-10.04	33.12	21.54	34.57	2.52	25
	2186.71	2248.60	2.83	58.60	34.52	59	2.62	25
Trigonelline	0.00	0.00	0.00	0.79	0.5	0.82	5.14	60
	4.97	5.01	0.75	1.18	1.02	1.4	6.64	20
	9.94	9.95	0.07	1.17	0.82	1.26	4.86	17
	19.88	20.28	2.03	1.25	0.33	1.07	2.93	13
	49.70	49.84	0.28	1.34	0.54	1.22	1.85	10
	198.80	192.22	-3.31	3.04	1.52	2.91	1.4	10
α-glucose	1190.00	1312.33	10.28	27.77	20.29	30.43	2.32	20
	1289.67	1335.71	3.57	66.90	40.59	68.05	5.09	20
	1314.59	1368.88	4.13	75.64	11.01	62.73	4.58	20
	1332.39	1428.19	7.19	97.03	46.98	92.11	6.45	20
	1389.34	1429.49	2.89	102.81	16.36	85.52	5.98	20
	2186.71	2233.29	2.13	47.26	21.72	44.28	1.98	20
β-glucose	1190.00	1205.47	1.30	37.34	34.07	45.72	3.79	20
	1289.67	1302.70	1.01	48.40	57.49	69.76	5.35	20
	1314.59	1316.82	0.17	55.75	28.15	53.52	4.06	20
	1332.39	1335.59	0.24	24.29	37.81	42.7	3.2	20
	1389.34	1400.04	0.77	22.19	26.28	31.92	2.28	20
	1439.18	1452.85	0.95	54.52	22.33	49.8	3.43	20
	1522.24	1331.65	-12.52	29.08	35.07	42.35	3.18	20
	2186.71	2299.76	5.17	73.15	43.52	73.9	3.21	20

[a] theoretical concentrations were calculated using OIV-approved methods.

[b] experimental concentrations are indicated as the mean of all measurements performed for each validation material (including triplicate of the five series).

Table S2. Concentrations of spiked standards used for calibration.

Compound	solvent	Spiked concentrations (mg/L)[b]
3-methylbutan-1-ol	H_2O	CF: 0; 101.5; 152.3; 203.1; 507.7
Acetic acid	H_2O	CF: 0; 78.7; 157.4; 236.1; 314.8
Glucose	H_2O	CF: 0; 203.0; 405.9; 608.9; 811.8
Caffeic acid	H_2O /EtOH[a]	CF: 0; 19.5; 29.3; 39.0; 97.6
Catechin	H_2O /EtOH	CF: 0; 48.9; 97.8; 146.7; 195.6
Epicatechin	H_2O /EtOH	CF: 0; 27.5; 55.0; 82.5; 110.0
Ethyl acetate	H_2O /EtOH	CF: 0; 29.6; 59.2; 88.7; 118.3
Ethly lactate	H_2O /EtOH	CF: 0; 24.5; 49.0; 73.5; 98.0
Formic acid	H_2O	CF & robustness: 0; 55.6; 83.5; 111.3; 278.2
		Linearity: 0; 900; 9,100; 11,500; 22,900
	H_2O /EtOH	Robustness: 0; 55.6; 83.5; 111.3; 278.2
	Wine	Robustness: 0; 50.5; 75.8; 101.0; 252.6
Fructose	H_2O	CF: 0; 296.8; 593.7; 890.5; 1187.3
Fumaric acid	H_2O	CF: 0; 29.3; 58.6; 87.9; 117.2
		Robustness: 0; 30.1; 45.1; 60.1
	H_2O /EtOH	Robustness: 0; 14.7; 29.3; 44.2; 58.7
	Wine	Robustness: 0; 32.4; 48.6; 64.8
Galacturonic acid	H_2O	CF: 0; 170.8; 341.7; 512.5; 683.4
Gallic acid	H_2O	CF: 0; 24.6; 49.2; 73.8; 98.4
Malic acid	H_2O	CF: 589.3; 1178.5; 1767.8; 2357.1
Shikimic acid	H_2O	CF: 0; 31.9; 63.9; 95.8; 127.8
Sorbic acid	H_2O	CF: 19.6; 29.4; 39.2; 98.1
	H_2O /EtOH	Robustness: 19.6; 29.4; 39.2; 98.1
	Wine	Robustness: 17.9; 26.9; 35.9; 89.7
Succinic acid	H_2O	CF: 0; 49.8; 99.7; 149.5; 199.3
Trigonelline	H_2O	CF: 0; 5.0; 9.9; 14.9; 19.8
Glycerol	H_2O	CF: 0; 3378.8; 6757.7; 10136.5; 13515.3
Methanol	H_2O	CF: 0; 49.5; 99.1; 148.6

[a] mixture water and ethanol (85/15 v/v).

[b] CF: samples used to determine the correction factor; Robustness: samples used to compare the matrix effect during calibration process; and Linearity: samples used to assess the method linearity.

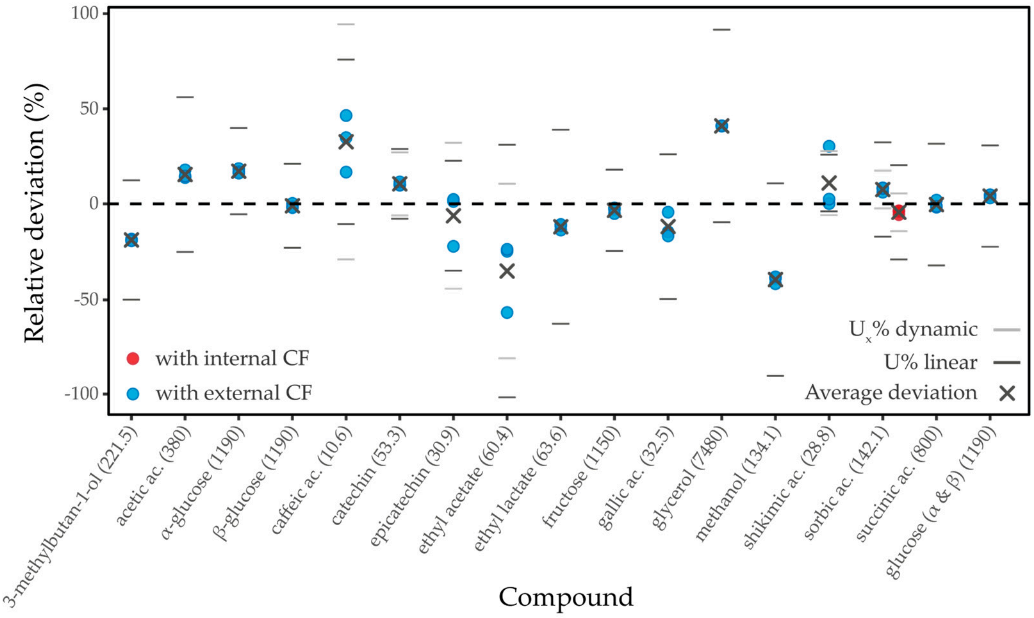

Figure S1. Relative deviation (%) between measured and theoretical values in the reference wine prepared with a 90:10 v/v mixture of wine and deuterated buffer solution.

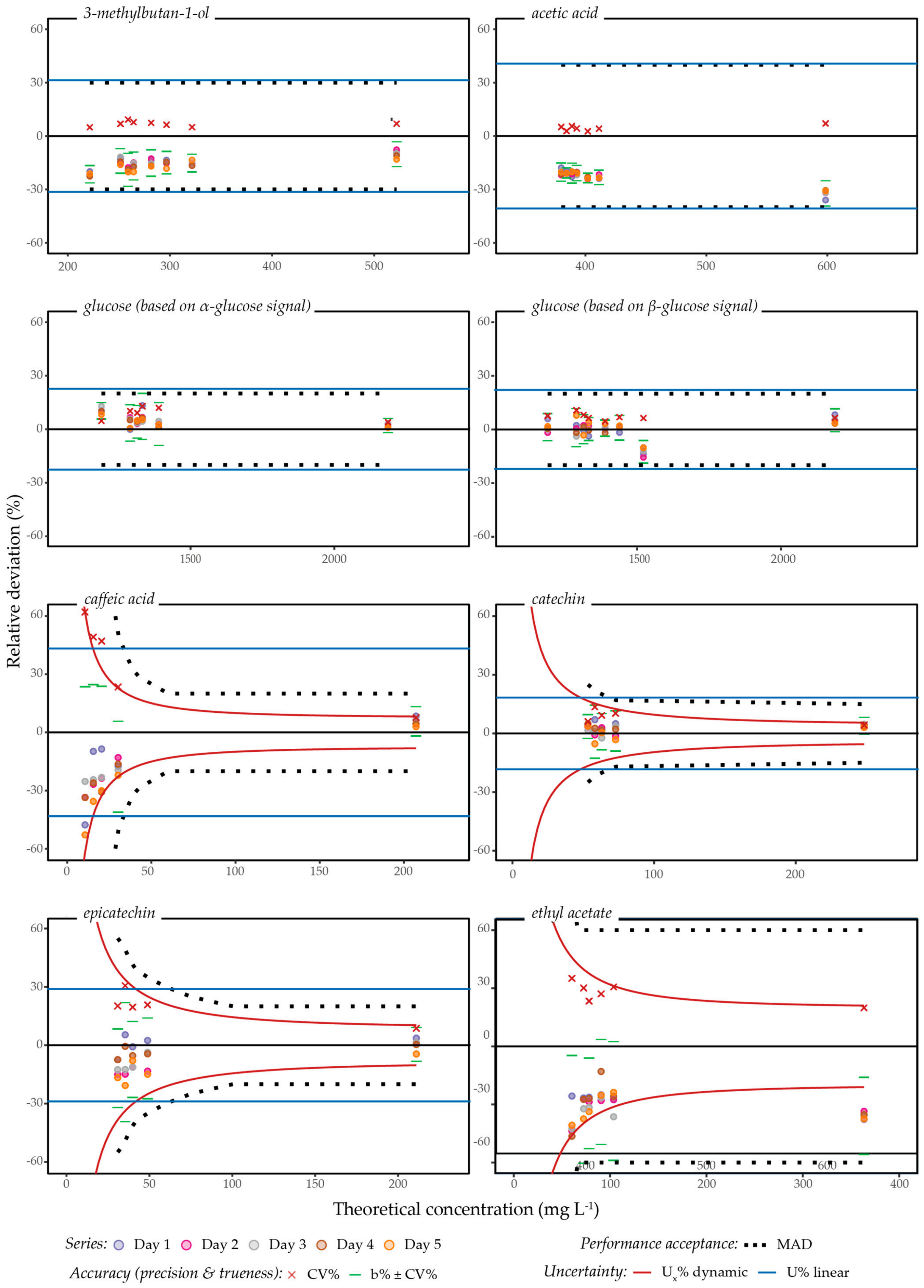

3-methylbutan-1-ol
acetic acid
glucose (based on α-glucose signal)
glucose (based on β-glucose signal)
caffeic acid
catechin
epicatechin
ethyl acetate
Relative deviation (%)
Theoretical concentration (mg L-1)
Series: Day 1 Day 2 Day 3 Day 4 Day 5
Accuracy (precision & trueness): CV% b% ± CV%
Performance acceptance: MAD
Uncertainty: Ux% dynamic U% linear

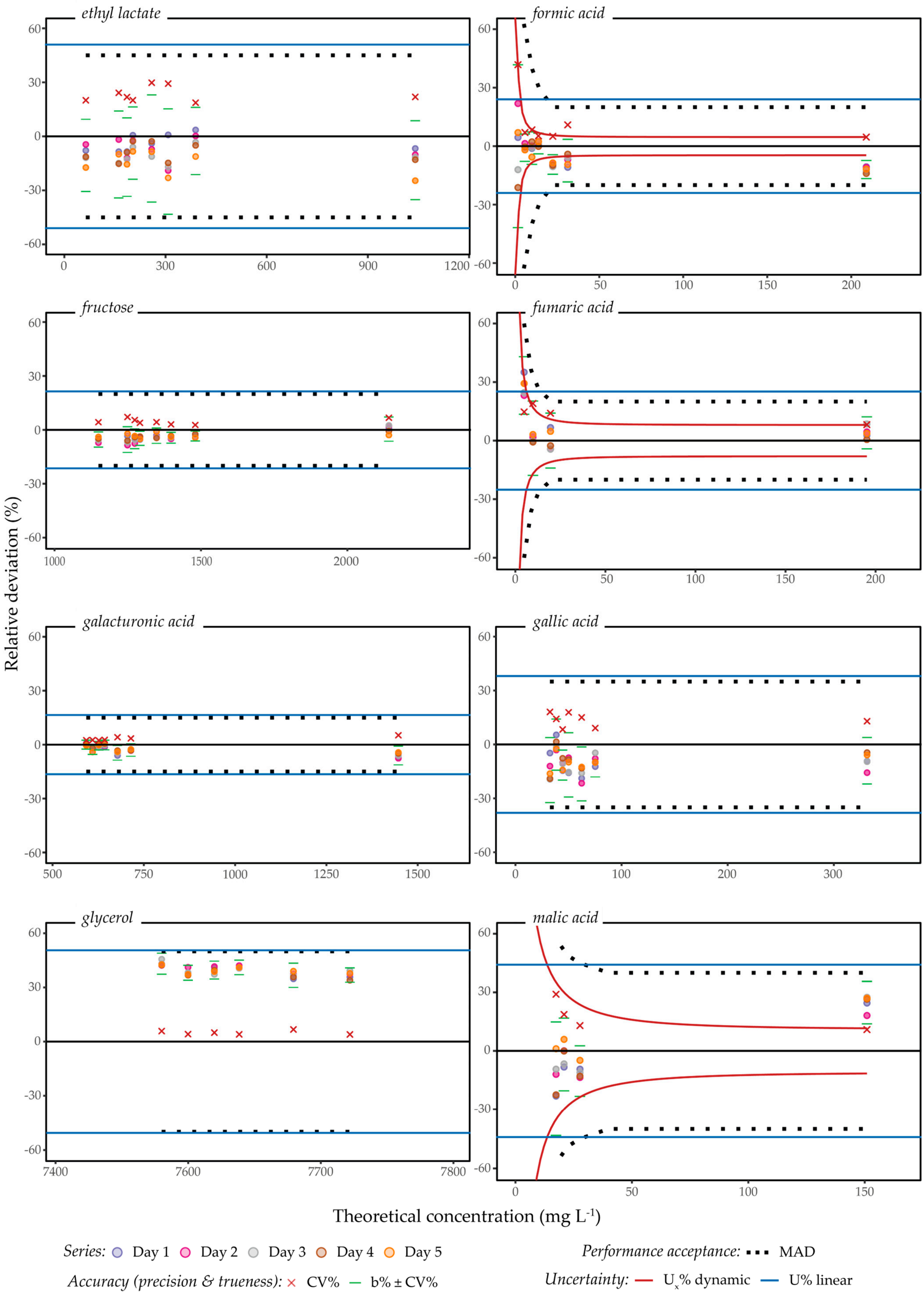

ethyl lactate
formic acid
fructose
fumaric acid
galacturonic acid
gallic acid
glycerol
malic acid
Relative deviation (%)
Theoretical concentration (mg L-1)
Series: Day 1 Day 2 Day 3 Day 4 Day 5
Accuracy (precision & trueness): CV% b% ± CV%
Performance acceptance: MAD
Uncertainty: Ux% dynamic U% linear

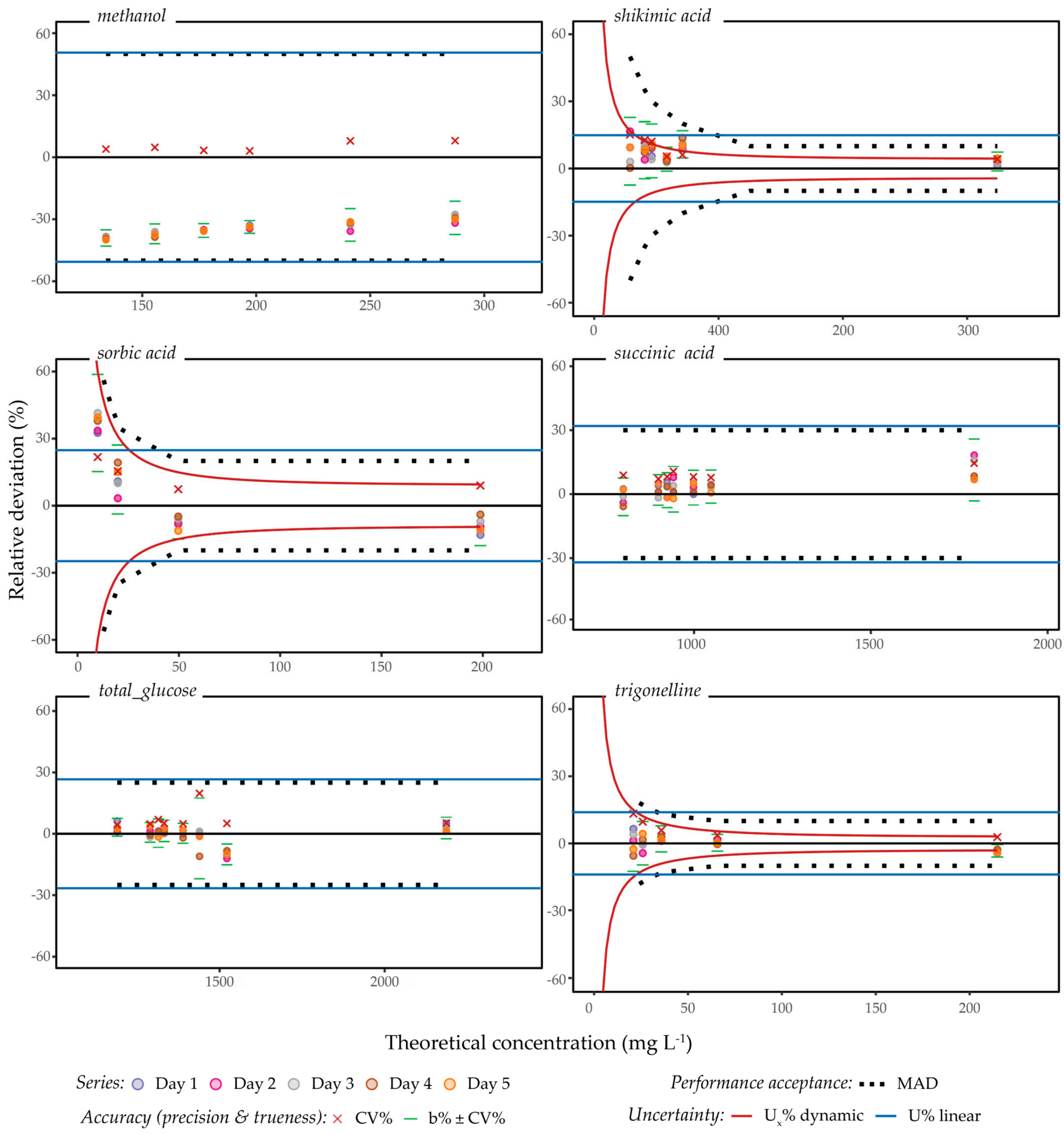

Figure S2. Accuracy profiles for all compounds obtained using the validation set.
